# Interaction of Polyphenols as Antioxidant and Anti-Inflammatory Compounds in Brain–Liver–Gut Axis

**DOI:** 10.3390/antiox9080669

**Published:** 2020-07-26

**Authors:** Amritpal Singh, Yu Fung Yau, Kin Sum Leung, Hani El-Nezami, Jetty Chung-Yung Lee

**Affiliations:** School of Biological Sciences, The University of Hong Kong, Pokfulam Road, Hong Kong, China; amritpal@connect.hku.hk (A.S.); hyfyau@connect.hku.hk (Y.F.Y.); sam612@connect.hku.hk (K.S.L.); elnezami@hku.hk (H.E.-N.)

**Keywords:** oxidative stress, inflammation, polyphenols, antioxidant

## Abstract

Oxidative stress plays an important role in the onset as well as the progression of inflammation. Without proper intervention, acute inflammation could progress to chronic inflammation, resulting in the development of inflammatory diseases. Antioxidants, such as polyphenols, have been known to possess anti-oxidative properties which promote redox homeostasis. This has encouraged research on polyphenols as potential therapeutics for inflammation through anti-oxidative and anti-inflammatory pathways. In this review, the ability of polyphenols to modulate the activation of major pathways of inflammation and oxidative stress, and their potential to regulate the activity of immune cells are examined. In addition, in this review, special emphasis has been placed on the effects of polyphenols on inflammation in the brain–liver–gut axis. The data derived from in vitro cell studies, animal models and human intervention studies are discussed.

## 1. Introduction

One of the main innate responses of the immune system is inflammation, which is an important non-specific response to any kind of injury and infection, such as physical wounds, toxins and tissue damage. It is a crucial response to the alteration of tissue integrity, to initiate healing and restore tissue homeostasis [1]. Several types of white blood cells, such as neutrophils and macrophages, and cytokines are involved in the inflammatory process. Cytokines play an enormous part in the inflammatory response and are mainly produced by helper T cells and macrophages [2]. They can be classified into pro-inflammatory cytokines, such as interleukin (IL)-1β and the tumor necrosis factor (TNF)-α, and anti-inflammatory cytokines, such as IL-4 and IL-10 [2]. The regulation and balance between the two types of cytokines is crucial for the immune system. An overproduction of pro-inflammatory cytokines could lead to autoimmune diseases and chronic inflammatory diseases, thus highlighting the need for anti-inflammatory cytokines to prevent chronic inflammatory conditions [2].

The inflammatory response is a multi-stage process which involves a triggering system, a sensor mechanism, signal transmission and the production of inflammatory mediators [1]. The inflammatory response could be triggered by various danger signals, which could be from exogenous, such as invasion by microorganisms, or endogenous sources, such as tissue damage. The exogenous and endogenous signaling molecules are termed pathogen-associated molecular patterns (PAMPs) and damage-associated molecular patterns (DAMPs) respectively [1]. PAMPs and DAMPs are sensed by a variety of pattern recognition receptors (PRRs) which include Toll-like receptors (TLRs), nucleotide-binding oligomerization domain (NOD)-like receptors (NLRs), C-type lectins and receptors for advanced glycation end-products (RAGE) [1]. The activation of PRR triggers intracellular signaling cascades, including kinases, such as mitogen-activated protein kinases (MAPKs), adaptors, the myeloid differentiation primary response protein 88 (MyD88), and transcription factors, such as the nuclear factor kappa B (NF-κB). Furthermore, the activation of NLR could trigger cytokine maturation, which are key to inflammation development, through inflammasomes. For instance, activated NLRP3 are associated to the adaptor protein ASC (apoptosis associated speck-like containing a CARD domain) and caspase-1 to form inflammasome, which promotes the conversion of pro-IL-1β and pro-IL-18 to mature IL-1β and IL-18, respectively [1]. Upon DAMP binding, TLR interacts with MyD88 which activates downstream signaling, resulting in NF-κB and activator protein-1 (AP-1) activation [3]. The signaling pathways mentioned above upregulate the expression of inflammatory mediators like cytokines for inflammation development.

It has been long known that significant oxidative stress could cause cellular damage and modification of genes, which triggers the inflammatory signaling cascade for the onset of inflammation in various inflammatory diseases [4]. As part of the inflammatory response, large amounts of reactive oxygen species (ROS) are generated, which could further promote oxidative stress and chronic inflammation if produced for lengthened periods [1,4]. Besides, several studies have pointed out that oxidants have a significant part in the activation of TLRs [5,6]. The studies have shown that the translocation of TLR4 to the cell membrane was upregulated after exposure to oxidants [5,6]. This enhances the responsiveness of cells to a danger signal for the onset of pro-inflammatory signaling pathways. One of the most discussed pathways in inflammation is the NF-κB pathway. It is a key regulator of inflammation due to its sensitivity to ROS. The activation of NF-κB could result from two pathways, the canonical and alternative pathways [1,7]. In both pathways, NF-κB is freed from its inhibitor, IκB, resulting in NF-κB translocation to the nucleus for the expression of target genes [1].

Under oxidative stress, the expression of antioxidant genes is upregulated, which is modulated by the nuclear factor erythroid 2-related factor 2 (Nrf2) [8]. Nrf2 activation is induced by ROS by the removal of its inhibitor, Kelch-like erythroid CNC homolog-associated protein 1 (Keap1), allowing the translocation of Nrf2 to the nucleus for the expression of genes involved in the antioxidant response [8].

As mentioned earlier, significant oxidative stress results in the propagation of inflammation, which illustrates the importance of redox balance in the resolution, and prevention of inflammation. Redox homeostasis is maintained by antioxidants, which could be from endogenous or exogenous (natural or synthetic) sources. The endogenous sources of antioxidants consist of enzymes such as glutathione peroxidase (GPx), superoxide dismutase (SOD) and catalase (CAT) [4]. Minerals such as zinc, selenium and copper are essential for the activation of the antioxidant enzymes as they are co-factors for these enzymes [9]. The natural exogenous antioxidants include ascorbic acid (vitamin C), α-tocopherol (vitamin E), carotenoids and flavonoids [4,9]. Antioxidants can scavenge free radicals which terminates the chain reaction of oxidation. Besides, they could prevent the initiation of a chain reaction by binding to transition metal ions that catalyze ROS generation [4,9]. As a result, antioxidants are able to reduce oxidative stress.

**Figure 1:**
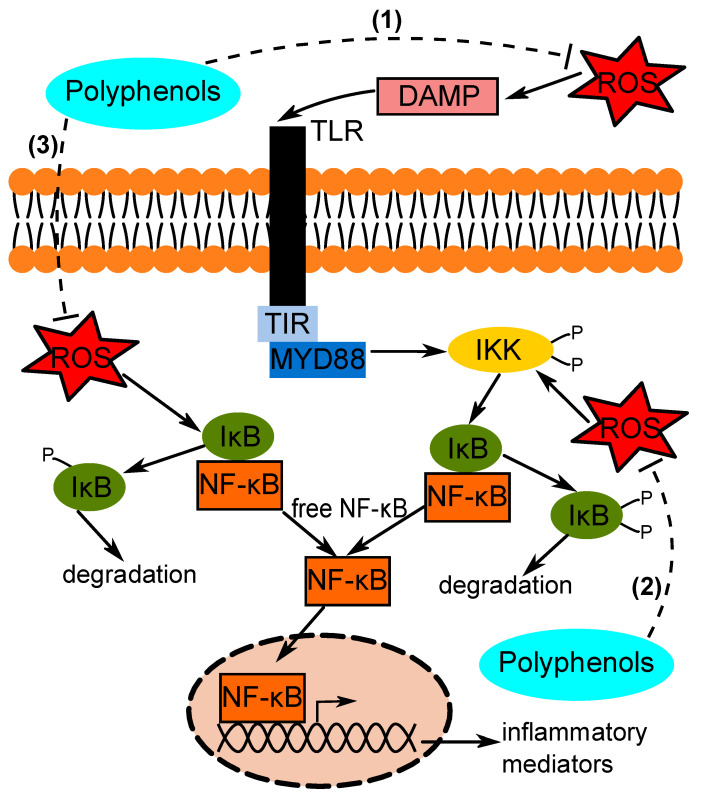
Potential mechanism of action of polyphenols in inflammation inhibition. Polyphenols may target the reactive oxygen species (ROS) to reduce oxidative stress. (1) ROS reduction could reduce the amount of damage-associated molecular patterns (DAMPs); (2) ROS reduction could also arrest phosphorylation of IκB kinase (IKK), which would block the dissociation of IκB from NF-κB; (3) ROS could directly phosphorylate IκB, which could be prevented by polyphenols. These pathways would inhibit nuclear translocation of NF-κB.

Polyphenols have been long known to be potent antioxidants. Polyphenols are found in various types of food, including fruits and vegetables, and can be classified into flavonoids and non-flavonoids [10]. Examples of flavonoids include anthocyanins, epigallocatechin gallate (EGCG) and curcumin, while an example of a non-flavonoid is resveratrol (RES) [10,11,12]. Because of their effects on oxidative stress, researchers have studied the effects of polyphenols in conditions with common underlying factors such as oxidative stress and inflammation. Polyphenols have been studied as potential anti-inflammatory agents in various inflammatory diseases, such as non-alcoholic fatty liver disease (NAFLD), inflammatory bowel disease and neurodegenerative diseases [10,11,12]. As illustrated in Figure 1, it is hypothesized that polyphenols would modulate the inflammatory signaling pathway via an antioxidant-based mechanism. It is expected that polyphenols would reduce oxidative stress, which would inhibit signal transduction for the production of pro-inflammatory mediators. The aim of this review will be to discuss the effects of polyphenol intervention in experimental and clinical settings on inflammation in specific organs, namely the brain, liver and gut, with reference to their antioxidant and anti-inflammatory properties.

## 2. Polyphenol Intervention in Brain Inflammation

Some of the most common brain disorders include neurodegenerative diseases such as Alzheimer’s disease and Parkinson’s disease [10,11,12,13]. Despite the exact causes of these diseases being unclear, these disorders have been linked to common underlying factors which are high levels of oxidative stress and inflammation [10,11,12,13]. Due to these reasons, the use of antioxidants as potential therapeutic agents has emerged. For instance, the effects of polyphenols have been studied on factors that are involved in disease progression. The findings of these studies in the last 15 years (2004–2019) are summarized in Table 1.

### 2.1. In Vitro Models of Polyphenol Treatment in Brain Inflammation

Among several in vitro studies, one group reported the inhibition of TLR4 signaling in cells treated with quercetin [14]. Besides, the expressions of TLR2 and TLR4 being reduced by quercetin, it further decreased the production of pro-inflammatory cytokines [15]. Furthermore, quercetin has been shown to attenuate the activity of inflammatory enzymes, including inducible nitric oxide synthase (iNOS) and cyclooxygenase (COX) [15]. In studies that tested anthocyanin-rich extracts in response to lipopolysaccharide (LPS) stimulation, a significant reduction in the production of TNF-α and IL-1β, and expressions of iNOS and COX-2 was reported [17,18,19,20,21]. A study on rat highly aggressively proliferating immortalized (HAPI) cells demonstrated similar results, but with no significant effect on iNOS expression [23]. In mouse microglial cells, anthocyanins reduced NO and TNF-α release and iNOS expression [22]. The reduced levels of pro-inflammatory mediators have been attributed by the modulation of inflammatory signaling pathways, since decreased levels of active p38 MAPK and NF-κB have been observed [17,18,19,20,21]. Besides microglial cells, anthocyanins testing on astrocytes has also revealed similar findings. For instance, rat astrocytes treated with anthocyanin-rich lingonberry extract lowered ROS production, suggesting an anti-oxidative mechanism [24]. In a human astrocyte study, the secretion of IL-6 was reduced at low doses of LPS and anthocyanins [16]. However, in the absence of LPS, IL-6 secretion increased when treated with high concentration of anthocyanin, suggesting antioxidant toxicity with a single compound supplementation [16]. Some studies have highlighted that the synergistic effects of mixed polyphenols provided better outcomes than single compound supplementation. Moreover, it was reported that the anti-inflammatory effects were more significant when the cells were treated with high concentrations of anthocyanins.

### 2.2. In Vivo Models of Polyphenol Treatment in Brain Inflammation

In a mouse model of Parkinson’s disease, the neuroprotective effects of grape seed and skin extract (GSSE), which is a mixture of polyphenolic compounds that is mostly comprised of flavonoids namely catechins, gallic acid, vanillin and 2,5-dihydroxybenzoic acid, were studied [25]. A reduction in ROS production, nuclear translocation of NF-κB p65 subunit and loss of SOD was observed [25]. RES inhibited TLR4, NF-κB and cytokine secretion in LPS and β-amyloid (Aβ)-mediated microglia neuroinflammation [26]. EGCG could attenuate TLR4 signaling in LPS-impaired adult hippocampal neurogenesis [27]. In addition, findings from anthocyanin treatment in LPS-induced mouse models have reported a decrease in several markers of oxidative stress and pro-inflammation. For example, levels of pro-inflammatory cytokines, such as TNF-α, IL-6 and IL-1β, were attenuated [28,29,30,31]. Furthermore, NF-κB activation was inhibited [28,29,30]. In one study, the expression of anti-inflammatory cytokines, such as IL-10, increased when the mice were pre-treated with anthocyanins [31]. Purple sweet potato extract showed anti-inflammatory effects in mice fed a high-fat diet (HFD), in which the expressions of iNOS, COX-2, TNF-α, IL-6 and IL-1β attenuated, and the levels of IL-10 increased [32]. Furthermore, the activation of p38 MAPK and NF-κB was inhibited [32]. Similarly, anthocyanin treatment in a rat middle cerebral artery occlusion/reperfusion (MCAO/R) model demonstrated decreased expressions of NF-κB, NLRP3 and pro-inflammatory cytokines [33].

For human studies in relation to polyphenol intervention, a few have been conducted. One study on the administration of RES was shown to decrease neuroinflammation in patients with Alzheimer’s disease [34]. The findings from in vitro and in vivo animal studies have demonstrated the benefits of polyphenols on brain inflammation. However, more human studies are required to validate the beneficial effects of polyphenols in brain-related inflammation.

## 3. Polyphenol Intervention in Liver Inflammation

One of most common liver diseases is NAFLD [35,36]. This disease could further progress to non-alcoholic steatohepatitis (NASH), and can eventually develop into hepatocellular carcinoma [35]. Similar to brain inflammation, the effects of polyphenol interventions in liver inflammation have been extensively researched. The findings of the effects of polyphenols on liver health in the last 15 years (2004–2019) are summarized in Table 2.

### 3.1. In Vitro Models of Polyphenol Treatment in Liver Inflammation

A few in vitro studies have illustrated the beneficial outcomes of polyphenols on oxidative stress and inflammation. Mouse macrophages pre-treated with rutin attenuated oxidative stress and *MCP-1*, *TNF-α*, *IL-6*, *IFN-γ*, *IL-1β* genes expressions [40]. In a study on human hepatocyte HepG2 cells, the glutamate cysteine ligase (GCL) activity and GSH level were improved with cyanidin-3-*O*-β-glucoside (C3G) treatment [39]. Moreover, theaflavins could reduce ROS generation in the same type of cell line [38]. Curcumin treatment also increased GCL activity and GSH level, and attenuated ROS production in glucose-induced hepatic stellate cells [37].

### 3.2. In Vivo Models of Polyphenol Treatment in Liver Inflammation

Much like the in vitro studies, several animal in vivo studies have demonstrated encouraging effects of polyphenol interventions. Quercetin has shown to improve thiobarbituric acid reactive substances (TBARS), GPx and CAT levels, and down-regulate TNF-α, IL-6 and COX-2 mRNA expressions in two mouse models [41,42]. Moreover, it was reported that the TLR4 protein concentration also decreased [42].

In mice fed with HFD, the expressions of TNF-α and MCP-1 genes were attenuated by rutin [40]. In another study, troxerutin increased GPx, SOD and GSH levels, which subsequently reduced ROS production [43]. Theaflavins supplementation lowered TBARS, ROS and TNF-α production in mice fed with a methionine and choline deficient high fat diet (HFD) [44]. Furthermore, the levels of MCP-1 and TNF-α were decreased with baicalein supplementation in HFD mice [45]. Oxidative stress could be reduced through the inhibition of macrophage infiltration when HFD mice are treated with RES [46]. In a few studies in which mice were given a methionine and choline-deficient diet, oxidative stress and inflammation were reduced with curcumin [47,48]. One of the studies reported the attenuation of ICAM-1, COX-2 and MCP-1 expressions and NF-κB signaling [47]. Another phenolic compound, silibinin, was shown to reduce iNOS expression, ROS production and NF-κB activation in mice fed with methionine and choline-deficient diet [49]. In addition, a similar model reported that silibinin supplementation improved GSH levels, and down-regulated IL-6 and TNF-α expressions [50].

Besides mouse models, polyphenol intervention has been studied in rat models. In rats fed with HFD, quercetin supplementation upregulated the expressions of Nrf2 and heme oxygenase 1 (HO-1), and down-regulated the expression of NF-κB [51]. In a similar model, mice supplemented with rutin had higher GPx expression and lower plasma MDA levels [52]. One study showed that, with EGCG supplementation, the GSH level was improved, whereas the plasma and liver MDA levels were reduced in rats fed with HFD [53]. Furthermore, EGCG could increase GPx and CAT activity, and attenuate iNOS, COX-2 and TNF-α expressions [54]. Similarly, genistein treatment decreased TNF-α, and plasma and liver MDA levels in HFD-fed rats [55]. In rats fed with a high cholesterol diet and naringenin, the attenuation of the production of pro-inflammatory cytokines, namely TNF-α, IL-6 and IL-1β, through the inhibition of the NF-κB pathway was also reported [56]. In fructose fed rats, RES increased SOD activity and Nrf2 and GSH levels [59]. Furthermore, RES reduced lipid peroxidation in the same model. The effects of coffee polyphenols have also been studied in a few rat models. The supplementation of coffee polyphenols ameliorated GSH/glutathione disulphide (GSSG) ratio, and attenuated TNF-α and IFN-γ expressions [57]. Besides, coffee polyphenols could reduce hepatic oxidative stress and steatosis in rats fed with HFD [58]. It was also reported that coffee polyphenols suppressed not only the expressions of pro-inflammatory cytokines, but also suppressed the expressions of anti-inflammatory cytokines, such as IL-4 and IL-10 [57]. 

A few of the polyphenols have also been subject to clinical testing, with the target group being the patients with NAFLD. For instance, supplementation of catechins decreased urinary F_2t_-isoprostane excretion in the treatment group that was given a higher dose [60]. Moreover, silymarin supplementation ameliorated the NASH score and serum oxidative stress [63]. A few studies have tested the effects of RES in NAFLD patients and have produced mixed results. Several studies observed an improvement in the inflammatory markers, such as TNF-α, cytokeratin 18 (CK-18), IL-6 and NF-κB [61,62]. On the other hand, one study reported the harmful effects of a high concentration of RES, in which the levels of enzymes, like alanine aminotransferase (ALT) and aspartate transaminase (AST) increased [64]. These findings suggest that polyphenols could prevent the progression of NAFLD to NASH. However, supplementation of a high dose could have adverse effects on liver health. 

## 4. Polyphenol Intervention in Gut Inflammation

Compared to the other organs that have been discussed in this review, polyphenol intervention in gut inflammation had not been a subject of profound research until recently. Most of the research has been on berry polyphenols and RES [65,66]. The interventions have targeted the models of inflammatory bowel disease, which is a term used to describe inflammatory disorders in the gut [66]. Some common examples of inflammatory bowel disease include Crohn’s disease and ulcerative colitis (UC) [66]. The findings of polyphenol interventions in vitro and in vivo studies in the last 15 years (2004–2019) are summarized in Table 3.

### 4.1. In Vitro Models of Polyphenol Treatment in Gut Inflammation

Much like the brain and liver, quite a few studies have been conducted to evaluate the effects of polyphenols on gut health. The amelioration of oxidative stress and inflammatory markers has been observed in several in vitro studies on human intestinal cell lines. For example, the expressions of TNF-α, IFN-γ-induced protein 10 (IP-10) and IFN-γ receptor 2 were inhibited by anthocyanins [67,68]. One study demonstrated the beneficial effects of RES in human intestinal Caco-2 cells treated with LPS. It was reported that RES pre-treatment suppressed COX-2 expression, prostaglandin E2 (PGE2) release and NF-κB activation [69]. In a similar study, iNOS and TLR4 expression, NF-κB activation and NO release were reduced but only in high RES concentration treatment groups [70]. The same results were obtained from RES treatment in human colon SW480 cells [70]. Moreover, RES upregulated HO-1 and GCL expression through the activation of the Nrf2 pathway and improved the GSH/GSSG ratio in human colon epithelial HT-29 cells [73]. Besides, ROS production, iNOS and COX-2 expression, and NO and PGE2 release could be attenuated with RES. However, it had no significant effect on the activation of NF-κB [72]. In Caco-2 cells stimulated by IL-1β or TNF-α, the phosphorylation of IκB could not be inhibited by RES, which allowed the activation of NF-κB [71]. This shows that stimulation by pro-inflammatory cytokines could trigger the inflammatory cascade even in the presence of an anti-inflammatory agent like RES.

### 4.2. In Vivo Models of Polyphenol Treatment in Gut Inflammation

In vivo animal models have also been used to better understand the impact of polyphenols. In a rat model of colitis induced by dextran sodium sulphate (DSS), blueberry powder reduced myeloperoxidase (MPO) activity and MDA concentration, but had an insignificant effect on MCP-1 level [90]. MPO activity is used to determine neutrophil infiltration, where a high activity would represent higher oxidative stress [65]. In another rat model of DSS-induced colitis, RES treatment decreased COX-2, PGE2 and NO levels. However, no significant change occurred in the TBARS level [98]. Another substance used to induce colitis in animal models is 2, 4, 6-trinitrobenzenesulfonic acid (TNBS). One study observed the attenuation of MPO activity, and VCAM-1, ICAM-1, MDA, NO and GSH levels with RES administration [91]. In addition to these effects, RES administration suppressed the expressions of MCP-1, cytokine-induced neutrophil chemoattractant 1 (CINC-1), TNF-α, IL-1β, IL-6 and IL-12 in rats with TNBS-induced colitis [92]. Pre-treatment of rats with RES lowered MPO activity, and IL-1β, PGE2 and prostaglandin D2 (PGD2) levels from TNBS-induced colitis [93]. In one study, even though the expression of certain inflammatory mediators was downregulated with RES treatment, the level PGE2 was increased [94]. Moreover, RES reduced colon MDA level and promoted GPx activity. Despite that, the MPO, SOD and CAT activities were unaffected [95]. In rats with methotrexate-induced or oxazolone-induced colitis, MPO activity was suppressed by RES administration [96,97]. Furthermore, the expression of pro-inflammatory cytokines, like IL-6, TNF-α and IL-1β, was decreased in a peptidoglycan-polysaccharide-induced colitis rat model [99]. 

Similar effects were demonstrated in mice models. Anthocyanins inhibited MPO activity, and the increase of pro-inflammatory cytokines in mice with TNBS-induced colitis. At the same time, the expression of the anti-inflammatory cytokine, IL-10, was upregulated [74]. Same effects were observed in both acute and chronic inflammatory conditions in a different study [77]. One study reported the suppression of inflammatory mediators and neutrophil infiltration, and the increase of CAT and SOD activity with blueberry extract. However, these effects were more prominent in the pre-treated group than the post-treated group [75]. Likewise, black raspberry extract attenuated TNF-α and IL-1β expressions, and NF-κB and COX-2 activity in mice with DSS-induced colitis. Yet, it had no significant effect on MDA and inflammatory cells infiltration [76]. Another study showed the inhibition of macrophages and neutrophils infiltration, and NF-κB nuclear translocation [78]. In a study on cranberry extract and dried cranberries treatment, dried cranberries attenuated MPO activity and pro-inflammatory cytokines production [79]. In mice that were given an HFD, cranberry extract downregulated the expression of inflammatory mediators but had an insignificant effect on MDA and SOD levels [80]. RES intervention has also been studied in various DSS-induced colitis mouse models. One study recorded a decrease in protein levels of iNOS, COX-2 and TNF-α [81]. Similar effects were observed in another study alongside an increase in IL-10 level [82]. In addition, a few studies showed an attenuation in the levels of pro-inflammatory cytokines and inflammatory enzymes with RES administration [83,88]. Moreover, RES supplementation reduced iNOS protein levels and NF-κB activation in colons of mice with DSS-induced colitis [87]. In mice with spontaneous chronic colitis, RES administration for 28 weeks decreased the levels of pro-inflammatory cytokines in the colon and serum [89]. On the other hand, RES had no significant impact on MPO activity and levels of TNF-α, PGE2, IL-6 and IL-10 in mouse models of DSS-induced colitis [84,86]. Although the MPO activity and the expressions of pro-inflammatory cytokines were downregulated, Yao et al. found the SOD and GPx activities to be suppressed as well [85]. 

Several studies have been conducted on polyphenol intervention in patients with UC. For instance, anthocyanins were reported to reduce TNF-α, IFN-γ and MCP-1 levels and NF-κB activation. In the same study, the levels of IL-22, IL-10 and IL-17A were elevated [68]. One study on subjects with mild to moderate UC showed a reduction in fecal calprotectin level with anthocyanins treatment, which suggests that neutrophil migration was lowered. However, an increase in disease activity was observed after the termination of the treatment [100]. Besides anthocyanins, RES has also been tested in subjects with UC. One study reported a decrease in high-sensitivity CRP and TNF-α levels, and the suppression of NF-κB activation [101]. Additionally, RES administration in patients with mild to moderate UC ameliorated plasma SOD activity and lessened plasma MDA level [102]. Even though there have been encouraging results from some clinical interventions, more clinical trials are required to consider polyphenols as potential therapeutics for gut inflammatory diseases.

## 5. Research Gap

Although polyphenols have demonstrated anti-inflammatory properties in vitro and in vivo animal studies, there is inconclusive evidence of their effects in humans. Currently, there is insufficient evidence to support the use of polyphenols as therapeutics in subjects with inflammatory diseases. There is a need for more human trials on polyphenol intervention to gain more conclusive evidence. Moreover, it is worth noting that the available human studies have only demonstrated symptom amelioration in subjects with inflammatory diseases. For instance, RES administration reduced inflammation in Alzheimer’s disease patients [34]. Other studies also reported the amelioration of symptoms with polyphenol supplementation in subjects with liver inflammatory diseases [60,61,62,63]. Furthermore, remission was observed from clinical interventions related to polyphenol administration in gut inflammatory disease patients [68,100,101,102]. Albeit these trials demonstrated beneficial effects, they could not demonstrate complete resolution of inflammation. As a result, it is rather premature to use polyphenols to treat inflammatory diseases.

In addition, a few studies have shown that there could be harmful effects associated with polyphenol treatment. For example, studies have reported an increase in pro-inflammatory cytokine secretion and NF-κB activation [16,71,94]. A study also reported the downregulation of antioxidant enzymes [85]. In another report, it showed RES supplementation resulted in the deterioration of liver health in patients with NAFLD [64]. Most of these adverse effects have been related to the administered dose of polyphenols. Therefore, further research on the appropriate dosage of polyphenols to produce beneficial effects and prevent adverse effects is required to reach a consensus.

## 6. Conclusions

Several in vitro and in vivo animal studies have demonstrated the antioxidant and anti-inflammatory effects of polyphenols in the brain–liver–gut axis. Polyphenols have been shown to target different stages of the inflammatory cascade to reduce the severity of inflammation. In general, the natural antioxidants seem to be more useful in the prevention of inflammation rather than in resolution. Although some antioxidants have had promising effects in vitro and animal studies, those results could not be extrapolated to human studies. As a result, further research is needed on polyphenol intervention in human trials, and on ways to improve the bioavailability and efficacy of polyphenols in subjects with inflammatory diseases.

## Figures and Tables

**Table 1:** Summary of the effects of polyphenols on brain inflammation in the last 15 years (2004–2019).

Model of Study	Agent	Effects	Reference
In vitro			
Human neuroblastoma SH-SY5Y cells (oxysterol induced)	Quercetin	↓ TLR4 signaling	[14]
Human PBMC (oxLDL-induced)	Quercetin	↓ TLR2 and TLR4 expressions, NF-κB activation, inflammatory enzymes activity	[15]
Human astrocytes (LPS-induced)	Anthocyanins	↓ IL-6 secretion (low LPS and anthocyanin dose); ↑ IL-6 secretion (high anthocyanin dose in LPS absence)	[16]
Mouse BV2 microglial cells (LPS-induced)	Blueberry extract	↓ NO and TNF-α release, iNOS and COX-2 expressions, NF-κB nuclear translocation	[17,18,19]
Mouse BV2 microglial cells (LPS-induced)	Anthocyanins	↓ NO, PGE2, TNF-α and IL-1β release, iNOS and COX-2 expressions, NF-κB nuclear translocation	[20,21]
Mouse microglial cells (LPS/IFN-γ-induced)	Anthocyanins	↓ NO and TNF-α release, iNOS expression	[22]
Rat HAPI microglial cells (LPS-induced)	Tart cherry extract	↓ NO and TNF-α release, COX-2 expression; ↔ iNOS expression	[23]
Rat astrocytes (LPS-induced)	Lingonberry extract	↓ ROS production	[24]
*Animal*			
Mouse model (PD)	GSSE	↓ ROS production, inflammatory markers	[25]
Mouse model (LPS and Aβ-induced microglia neuroinflammation)	RES	↓ TLR4, NF-κB and cytokine secretion	[26]
Mouse model (LPS-impaired adult hippocampal neurogenesis)	EGCG	↓ TLR4 signaling	[27]
Mouse model (LPS-treated)	Anthocyanins	↓ NF-kB, TNF-α, and IL-1β levels	[28]
Mouse model (LPS-treated)	PSPC	↓ TNF-α, IL-6 and IL-1β overproduction, NF-kB activation	[29]
Mouse model (LPS-treated)	Anthocyanins	↓ ROS production, NF-kB activation, TNF-α, and IL-1β levels	[30]
Mouse model (LPS-treated)	Anthocyanins	↓ TNF-α, and IL-1β increase; ↑ IL-10 expression	[31]
Mouse model (high-fat diet)	PSPC	↓ iNOS, COX-2, TNF-α, IL-1β and IL-6 expressions, p38 MAPK and NF-kB activation; ↑ IL-10 levels	[32]
Rat model (MCAO/R)	Anthocyanins	↓ TNF-α, IL-6 and IL-1β levels, NF-kB and NLRP3 expressions	[33]
*Human*			
Subjects with AD	RES	↓ plasma pro-inflammatory markers	[34]

↑: increase; ↓: decrease; ↔: insignificant change; Aβ: beta-amyloid; AD: Alzheimer’s disease; COX: cyclooxygenase; EGCG: epigallocatechin gallate; GSSE: grape seed and skin extract; IFN-γ: interferon gamma; IL: interleukin; iNOS: inducible nitric oxide synthase; LDL: low-density lipoprotein; LPS: lipopolysaccharide; MAPK: mitogen-activated protein kinase; MCAO/R: middle cerebral artery occlusion/reperfusion; NF-κB: nuclear factor kappa B; NLRP: NOD-like receptor protein; NO: nitric oxide; ox: oxidized; PBMC: peripheral blood mononuclear cell; PD: Parkinson’s disease; PGE2: prostaglandin E2; PSPC: purple sweet potato color; RES: resveratrol; ROS: reactive oxygen species; TLR: toll-like receptor; TNF-α: tumor necrosis factor alpha.

**Table 2:** Summary of the effects of polyphenols on liver inflammation in the last 15 years (2004–2019).

Model of Study	Agent	Effects	Reference
*In vitro*			
Hepatic stellate cells (glucose-induced)	Curcumin	↓ ROS production; ↑ GCL activity, GSH level	[37]
Human HepG2 cells (fatty acid-induced)	Theaflavins	↓ ROS production	[38]
Human HepG2 cells (glucose-induced)	C3G	↓ ROS production; ↑ GCL activity, GSH level	[39]
Mouse macrophage cells (palmitic acid-induced)	Rutin	↓ ROS production, *MCP-1*, *TNF-α*, *IL-6*, *IFN-γ*, *IL-1β* genes expressions	[40]
*Animal*			
Mouse model (Western diet)	Quercetin	↓ TBARS, TG and TNF-α levels; ↑ GPx and CAT levels	[41]
Mouse model (MCD)	Quercetin	↓ TLR4 protein concentration, TNF-α, IL-6 and COX-2 mRNA expressions	[42]
Mouse model (HFD)	Rutin	↓ *TNF-α and Mcp1* gene expressions	[40]
Mouse model (HFD)	Troxerutin	↓ ROS levels; ↑ GPx, GSH and SOD levels	[43]
Mouse model (MCDHFD)	Theaflavins	↓ TBARS level, ROS production, TNF-α expressions	[44]
Mouse model (HFD)	Baicalein	↓ MCP-1 and TNF-α levels	[45]
Mouse model (HFD)	RES	↓ macrophage infiltration	[46]
Mouse model (MCD)	Curcumin	↓ ICAM-1, COX-2 and MCP-1 expressions, NF-κB signalling	[47]
Mouse model (MCD)	Curcumin	↓ ROS production	[48]
Mouse model (MCD)	Silibinin	↓ ROS production, iNOS expression, NF-κB activation	[49]
Mouse model (MCD)	Silibinin	↓ Il-6 and TNF-α expressions; ↑ GSH level	[50]
Rat model (HFD)	Quercetin	↓ NF-κB expression; ↑ Nrf2 and HO-1 expressions	[51]
Rat model (HFD)	Rutin	↓ plasma MDA; ↑ GPx expression	[52]
Rat model (HFD)	EGCG	↓ plasma and liver MDA; ↑ GSH level	[53]
Rat model (HFD)	EGCG	↓ iNOS, COX-2 and TNF-α expressions; ↑ GPx and CAT activity	[54]
Rat model (HFD)	Genistein	↓ TNF-α and plasma and liver MDA levels	[55]
Rat model (HCD)	Naringenin	↓ ROS production, TNF-α, IL-6, IL-1β and iNOS expressions	[56]
Rat model (HFD)	Coffee polyphenols	↓ TNF-α, IFN-γ, IL-4 and IL-10 expressions; ↑ GSH/GSSG ratio,	[57]
Rat model (HFD)	Coffee polyphenols	↑ GST expression	[58]
Rat model (fructose-fed)	RES	↓ TBARS level; ↑ SOD activity, Nrf2 and GSH levels	[59]
*Human*			
Subjects with NAFLD	Catechins	↓ urinary F_2t_-isoprostane excretion (high dose)	[60]
Subjects with NAFLD	RES	↓ inflammatory markers (TNF-α, CK-18, FGF-21)	[61]
Subjects with NAFLD	RES	↓ inflammatory markers (IL-6, hs-CRP, NF-κB)	[62]
Subjects with NAFLD	Silymarin	↓ NASH score and serum oxidative stress	[63]
Subjects with NAFLD	RES	↑ ALT and AST levels (high dose)	[64]

↑: increase; ↓: decrease; ALT: alanine aminotransferase; AST: aspartate transaminase; C3G: cyanidin-3-glucoside; CAT; catalase; CK: cytokeratin; COX: cyclooxygenase; EGCG: epigallocatechin gallate; FGF: fibroblast growth factor; GCL: glutamate-cysteine ligase; GPx: glutathione peroxidase; GSH: reduced glutathione; GSSG: oxidized glutathione; GST: glutathione s-transferase; HFD: high fat diet; HO: heme oxygenase; hs-CRP: high sensitivity C-reactive protein; ICAM: intercellular adhesion molecule; IFN-γ: interferon gamma; IL: interleukin; iNOS: inducible nitric oxide synthase; MCD: methionine-choline deficient; MCDHFD: methionine-choline deficient high fat diet; MCP: monocyte chemoattractant protein; MDA: malondialdehyde; NAFLD: non-alcoholic fatty liver disease; NASH: non-alcoholic steatohepatitis; NF-κB: nuclear factor kappa B; Nrf: nuclear factor erythroid 2-related factor; RES: resveratrol; ROS: reactive oxygen species; SOD: superoxide dismutase; TBARS: thiobarbituric acid reactive substances; TG: triglyceride; TLR: toll-like receptor; TNF-α: tumour necrosis factor alpha.

**Table 3:** Summary of the effects of polyphenols on gut inflammation in the last 15 years (2004-2019).

Model of Study	Agent	Effects	Reference
In vitro			
Human colon epithelial cells (cytokine-treated)	Anthocyanins	↓ IP-10 and TNF-α expression	[67]
Human monocytic THP-1 cells (IFN-γ-treated)	Anthocyanins	↓ IFN-γ receptor 2 expression	[68]
Human intestinal Caco-2 cells (LPS-treated)	RES	↓ COX-2 expression, PGE2 release, NF-κB activation	[69]
Human intestinal Caco-2 cells (LPS-treated)	RES	↓ iNOS and TLR4 expression, NF-κB activation, NO release (high dose)	[70]
Human intestinal Caco-2 cells (IL-1β-treated)	RES	↑ NF-κB activation, p-IκB/IκB ratio, IL-8 production	[71]
Human intestinal Caco-2 cells (TNF-α-treated)	RES	↑ NF-κB activation	[71]
Human colon epithelial HT-29 cells (cytokine-treated)	RES	↓ ROS production, iNOS and COX-2 expression, NO and PGE2 release; ↔ NF-κB activation	[72]
Human colon epithelial HT-29 cells (cytokine-treated)	RES	↑ HO-1 and GCL expression, Nrf2 activation, GSH:GSSG ratio	[73]
Human colon SW480 cells (LPS-treated)	RES	↓ iNOS and TLR4 expression, NF-κB activation, and NO release (high dose)	[70]
*Animal*			
Mouse model (TNBS-induced colitis)	Anthocyanins	↓ MPO activity, IL-12, TNF-α and IFN-γ increase, NO production; ↑ IL-10 expression	[74]
Mouse model (DSS-induced colitis)	Blueberry extract	↓ COX-2, iNOS, IFN-γ and IL-1β expression, NF-κB activation, neutrophil infiltration, MDA and serum PGE2 levels; ↑ CAT and SOD activity	[75]
Mouse model (DSS-induced colitis)	Black raspberry powder	↓ TNF-α and IL-1β expression, NF-κB and COX-2 activity; ↔ RNS and MDA levels, inflammatory cells infiltration	[76]
Mouse model (DSS-induced colitis)	Anthocyanins	↓ TNF-α and IFN-γ secretion	[77]
Mouse model (DSS-induced colitis)	Black raspberry powder	↓ macrophages and neutrophils infiltration, NF-κB translocation	[78]
Mouse model (DSS-induced colitis)	Cranberry extract or dried cranberries	↓ MPO activity, TNF-α and IL-1β expression	[79]
Mouse model (high fat diet)	Cranberry extract	↓ COX-2 and TNF-α expression, LPS level; ↔ MDA and SOD levels	[80]
Mouse model (DSS-induced colitis)	RES	↓ iNOS, COX-2 and TNF-α levels	[81]
Mouse model (DSS-induced colitis)	RES	↓ iNOS and COX-2 expression, TNF-α and IL-1β levels; ↑ IL-10 level	[82]
Mouse model (DSS-induced colitis)	RES	↓ IL-6, TNF-α, IFN-γ and IL-1β levels, COX-1 and COX-2 expression	[83]
Mouse model (DSS-induced colitis)	RES	↔ MPO activity and TNF-α level	[84]
Mouse model (DSS-induced colitis)	RES	↓ MPO, SOD and GPx activity, MDA level, TNF-α, IFN-γ and IL-8 expression	[85]
Mouse model (DSS-induced colitis)	RES	↔ MPO activity, PGE2, IL-6 and IL-10 levels	[86]
Mouse model (DSS-induced colitis)	RES	↓ iNOS level, NF-κB and IκB activation	[87]
Mouse model (DSS-induced colitis)	RES	↓ TNF-α level, COX-2 and IL-6 expression	[88]
Mouse model (Spontaneous chronic colitis)	RES	↓ IL-6, IL-12, TNF-α, IFN-γ and IL-1β levels	[89]
Rat model (DSS-induced colitis)	Blueberry powder	↓ MPO activity, MDA concentration; ↔ MCP-1 and GRO/CINC-1 levels	[90]
Rat model (TNBS-induced colitis)	RES	↓ MPO activity, VCAM-1, ICAM-1, MDA, NO and GSH levels	[91]
Rat model (TNBS-induced colitis)	RES	↓ MPO activity, GSH level, ICAM-1, MCP-1, CINC-1, TNF-α, IL-1β, IL-6 and IL-12 expression	[92]
Rat model (TNBS-induced colitis)	RES	↓ MPO activity, IL-1β, PGE2 and PGD2 levels	[93]
Rat model (TNBS-induced colitis)	RES	↓ MPO activity, TNF-α level, COX-1, COX-2 and NF-κB p65 expression; ↑ PGE2 level; ↔ PGD2 level	[94]
Rat model (TNBS-induced colitis)	RES	↓ MDA level; ↑ GPx activity; ↔ MPO, SOD, CAT activities	[95]
Rat model (Methotrexate-induced colitis)	RES	↓ MDA and GSH levels, MPO expression	[96]
Rat model (Oxazolone-induced colitis)	RES	↓ MPO activity	[97]
Rat model (DSS-induced colitis)	RES	↓ COX-2, PGE2 and NO levels; ↔ TBARS level	[98]
Rat model (PG-PS-induced colitis)	RES	↓ IL-6, TNF-α and IL-1β expression	[99]
*Human*			
Subjects with UC	Anthocyanins	↓ TNF-α, IFN-γ and MCP-1 levels, NF-κB activation; ↑ IL-22, IL-10 and IL-17A levels	[68]
Subjects with mild to moderate UC	Anthocyanins	↓ faecal calprotectin level and Riley Index	[100]
Subjects with UC	RES	↓ hs-CRP, TNF-α levels, PBMC NF-κB activation; ↑ IBDQ-9 score; ↔ SCCAI score	[101]
Subjects with mild to moderate UC	RES	↓ MDA level; ↑ SOD activity, IBDQ-9 score; ↔ SCCAI score	[102]

↑: increase; ↓: decrease; ↔: insignificant change; CAT; catalase; CINC: cytokine-induced neutrophil chemoattractant; COX: cyclooxygenase; DSS: dextran sodium sulphate; GCL: glutamate-cysteine ligase; GPx: glutathione peroxidase; GSH: reduced glutathione; GSSG: oxidized glutathione; HO: heme oxygenase; hs-CRP: high sensitivity C-reactive protein; ICAM: intercellular adhesion molecule; IFN-γ: interferon gamma; IL: interleukin; IP: IFN-γ-induced protein; iNOS: inducible nitric oxide synthase; LPS: lipopolysaccharide; MCP: monocyte chemoattractant protein; MDA: malondialdehyde; MPO: myeloperoxidase; NF-κB: nuclear factor kappa B; NO: nitric oxide; Nrf: nuclear factor erythroid 2-related factor; PBMC: peripheral blood mononuclear cell; PG-PS: peptidoglycan-polysaccharide; PGD2: prostaglandin D2; PGE2: prostaglandin E2; RES: resveratrol; RNS: reactive nitrogen species; ROS: reactive oxygen species; SOD: superoxide dismutase; TBARS: thiobarbituric acid reactive substances; TLR: toll-like receptor; TNBS: 2, 4, 6-Trinitrobenzenesulfonic acid; TNF-α: tumour necrosis factor alpha; UC: ulcerative colitis; VCAM: vascular cell adhesion molecule.
